# Spontaneous Haemothorax in a Patient with COVID-19

**DOI:** 10.1155/2022/8275326

**Published:** 2022-07-18

**Authors:** Shalini A. Mohan, Zharif Sufyaan Fadzaily, S. H. Abdullah.Hashim

**Affiliations:** ^1^Department of Internal Medicine, Ampang Hospital, Ampang, Selangor, Malaysia; ^2^Infectious Disease Unit, Department of Internal Medicine, Ampang Hospital, Ampang, Selangor, Malaysia

## Abstract

The global pandemic of COVID-19 is caused by SARS-CoV-2 virus. We continue to discover the wide spectrum of complications associated with COVID-19. Some well-known complications include pneumonia, acute respiratory distress syndrome, pneumothorax, disseminated intravascular coagulation (DIC), chronic fatigue, multiorgan dysfunction, and long COVID-19 syndrome. We report a rare case of a 51-year-old man with severe COVID-19 pneumonia who developed haemorrhagic shock secondary to spontaneous haemothorax after 17 days of hospitalisation. Clinicians should be aware of such occurrence, and hence, high clinical suspicion, prompt recognition of signs and symptoms of shock, and adequate resuscitation will improve the outcomes of patients.

## 1. Introduction

The severe acute respiratory syndrome coronavirus 2 (SARS-CoV-2) is the novel virus leading to the global pandemic of COVID-19. The most common manifestations of COVID-19 include fever, cough, and dyspnoea [1]. Endothelial dysfunctions, thrombosis, complement activation, cytokine release, and dysregulated inflammation are some mechanisms proposed for the pathogenesis of COVID-19 [2, 3]. Spontaneous haemothorax in nonventilated COVID-19 patients is an extremely rare occurrence and has only been described in another case report secondary to apical bullae rupture [4]. Here, we report a case of spontaneous haemothorax causing haemorrhagic shock in a COVID-19 patient with no history of trauma, iatrogenic injuries, barotrauma, or vascular malformation.

## 2. Case Presentation

A 51-year-old gentleman, an active smoker with no reported medical or surgical history, presented to our hospital's emergency room for fever, shortness of breath, and runny nose of five days duration with an oxygen saturation of 80% on room air. He was afebrile and normotensive but tachycardic with a heart rate of 130 beats/minute and was in respiratory distress requiring oxygen supplementation at 15 L/minute. He denied any history of trauma.

The blood gas analysis on supplementary oxygen 15 L/minute showed type 1 respiratory failure. Pulmonary auscultation found bilateral lower zone crackles. Chest radiography showed bilateral ground glass opacities especially basal. The remainder of the physical examination was unremarkable. The nasopharyngeal (NP) and oropharyngeal (OP) swabs taken in the emergency department for SARS-CoV-2 were positive.

The biochemical results showed features of hyperinflammation evidenced by absolute lymphocyte count: 0.7 K/L, an elevated level of C-reactive protein at 232 mg/L, ferritin of 4513.5 *μ*g/L, D-dimer of 1.52 *μ*g/mL, and lactate dehydrogenase (LDH) of 832 U/L.

**Figure 1:**
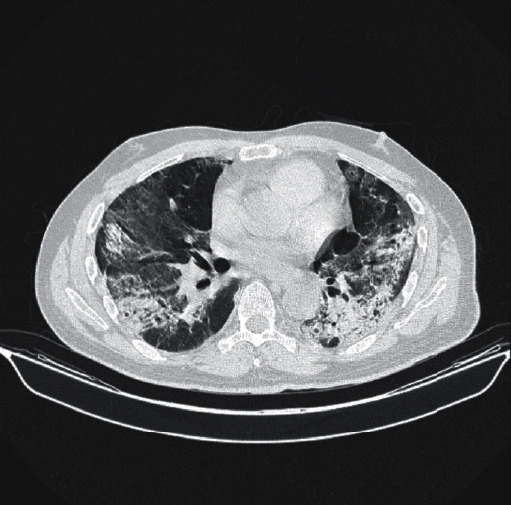
HRCT illustrating severe organising pneumonia with fibrotic-like changes.

A high-resolution chest computed tomography (HRCT) with pulmonary angiography acquisitions (CTPA) on day 26 of illness showed features typical of COVID-19 pneumonia with evidence of ground glass opacities of central and peripheral distribution in all lung lobes (Figure 1). No evidence of pulmonary artery thrombosis was found. He was on a prophylactic dose of subcutaneous enoxaparin since admission.

**Figure 2:**
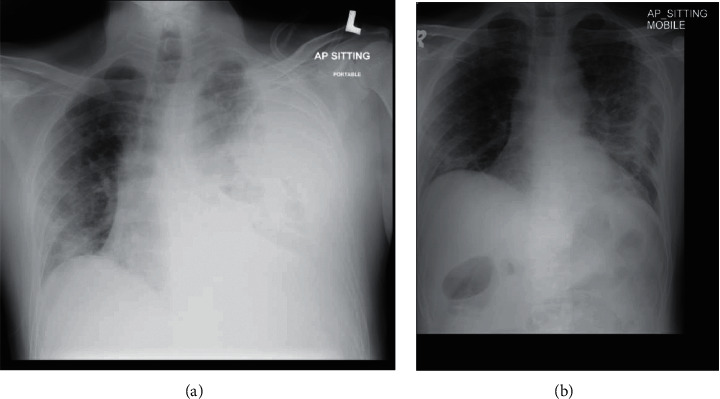
(a) A massive left sided pleural effusion with right mediastinal shift. (b) A marked improvement in left haemothorax.

**Figure 3:**
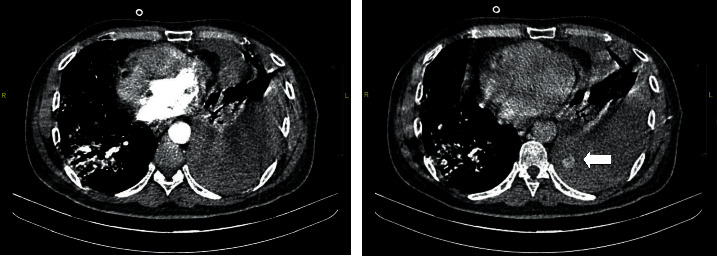
CT angiography of the thorax demonstrating a streak of contrast extravasation arising from 4^th^ intercostal space on portovenous phase with further pooling of contrast into pleural cavity at a 3-minute delayed phase (shown by the arrow).

On day 28 of illness, the patient complained of acute onset chest pain and was clinically pale, tachycardic, and hypotensive with a mean arterial pressure (MAP) of 65 mmHg. He also required an increased oxygen supplementation at 60 L/minute. There was a significant drop of haemoglobin level by 4 g/dL (13–8.9 g/dL) with no evidence of haematuria or gastrointestinal bleeding. He had a normal coagulation profile and creatinine clearance of 89 mL/min. Repeated chest radiograph showed massive left sided pleural effusion with right mediastinal shift (Figure 2(a)). We proceeded with an ultrasound guided intercostal drainage, yielding 500 mL fresh blood. A subsequent CT angiography thorax confirmed a massive left haemothorax, causing mediastinal shift with contrast extravasation in keeping with active bleeding arising from the left fourth intercostal vessel (Figure 3). There was no evidence of pulmonary artery aneurysm.

In view of haemorrhagic shock, he was transfused a total of five units of packed cells, two units of platelets, four units of fresh frozen plasma, and six units of cryoprecipitate. Our patient also required low dose inotropic support for four days.

Cardiothoracic surgery was consulted for angioembolization and watchful waiting was recommended as there was a marked reduction in the pigtail drainage. The intercostal pigtail catheter was removed after one week. Repeated chest radiograph showed a marked improvement in left haemothorax (Figure 2(b)). He was weaned off his oxygen support, highest requirement being high flow nasal cannula at 60 L/min to room air over a span of two weeks.

Our patient was discharged well after one month of hospitalisation.

## 3. Discussion

Since its discovery in Wuhan, China, in December 2019, the number of viral pneumonias caused by the coronavirus is rising dramatically and has quickly spread all over the world.

The main symptoms of the SARS-CoV-2 infection described in a descriptive study by Chen et al. in Wuhan were fever, cough, dyspnoea, and arthralgia [1]. Other cardiovascular and gastrointestinal symptoms, such as chest pain, diarrhoea, nausea, and vomiting, were also reported.

The pathophysiology of COVID-19 is linked to vascular damage and host immune response dysregulation. This leads to cytokine storm which correlates with clinical severity of the disease [2, 3].

Haemothorax is defined as a collection of blood in the pleural space, usually due to lesions of the lung parenchyma, pleura, chest wall, mediastinum, or abdomen [5]. Haemothorax generally develops secondary to trauma and it is rarely spontaneous. Spontaneous haemothorax is extremely rare in COVID-19, and only few cases have been reported so far [4, 6].

Vascular pathologies, necrotising infections, connective tissue disorders, pleural diseases, malignancy, and bleeding disorders are some causes of spontaneous haemothorax [7]. Postulated causes of haemothorax in COVID-19 include pulmonary artery aneurysm [6], barotrauma [8], and necrotising pneumonia [9].

Research into COVID-19 demonstrated inflammatory and vasculitic changes involving pulmonary vasculatures, skin, and Kawasaki-like disease phenomenon [10]. Guven et al. reported a case of spontaneous haemothorax in a young patient following mechanical ventilation. Prolonged mechanical ventilation possibly led to the separation of pleura due to large variation in pleural pressure, resulting in haemothorax [8]. Our patient, however, did not have any barotrauma as a result of mechanical ventilation.

Pulmonary artery aneurysm caused by vasculitic changes was also another postulated cause for spontaneous haemothorax for patients with COVID-19 as reported by Desnos et al. [6] This patient, however, showed no evidence of pulmonary artery aneurysm in the CT angiography of the thorax.

Our patient also showed no evidence of necrotising pneumonia or bullae rupture, two other well described causes of spontaneous haemothorax in patients with COVID-19 reported by Jung et al. and Brogna et al., respectively [4, 9].

In this case, the patient's spontaneous haemothorax from active bleeding of the left fourth intercostal artery is potentially due to vasculitic changes, resulting in haemorrhagic shock as he had no history of trauma, iatrogenic injuries, barotrauma, or vascular malformation. As research related to COVID-19 and its complications emerge, it remains to be seen if further spontaneous haemorrhagic events will be associated with COVID-19 disease.

A CT scan of the thorax remains the modality of choice for visualising the severity of COVID-19 pneumonia and its complications. A CTPA can also rule out pulmonary embolism and reveal vascular malformations or any source of arterial bleeding [5, 11].

For haemodynamically stable patients, intercostal drainage via a pigtail or tube thoracostomy should suffice. Most cases of haemothorax will resolve with tube thoracostomy. If residual blood remains in the thoracic cavity postthoracostomy and medical therapy including fibrinolytics has failed, then surgical intervention is required. Fibrinolytics are infused into the pleural space, disrupting the haemothorax and facilitates further drainage. Video-assisted thoracoscopy (VATS) has emerged as the surgical intervention of choice for haemodynamically unstable patients as well as residual haemothorax. However, should all previous attempts to resolve the haemothorax fail, then open thoracotomy is indicated [12].

In the event of persistent bleeding from posterior intercostal arteries, haemostasis may be difficult to achieve due to limited exposure at the posterior ribs space. Transarterial embolisation remains the endovascular therapeutic option of choice to control such intrathoracic haemorrhage [13].

## 4. Conclusion

This case, while being uncommon, highlights a potentially life-threatening complication of COVID-19. COVID-19 patients have an increased likelihood of thromboembolic events, and a concomitant spontaneous haemorrhage imposes a challenge to treating physicians, especially while choosing an anticoagulation strategy. Further research into coagulopathy associated with COVID-19 infection may be helpful. High clinical suspicion, early recognition of signs of shock, and adequate resuscitation play a pivotal role in improving the outcomes of patients.

## Data Availability

The data used to support the findings of this study are available from the corresponding author upon request.
